# Suicidal Ideation and Attempts Among Youth With Physical-Mental Comorbidity in Canada: Proposal for an Epidemiological Study

**DOI:** 10.2196/57103

**Published:** 2024-07-04

**Authors:** Mark A Ferro, Christy K Y Chan, Dillon T Browne, Ian Colman, Joel A Dubin, Laura Duncan

**Affiliations:** 1 School of Public Health Sciences University of Waterloo Waterloo, ON Canada; 2 Department of Psychology University of Waterloo Waterloo, ON Canada; 3 School of Epidemiology and Public Health University of Ottawa Ottawa, ON Canada; 4 Department of Statistics and Actuarial Science University of Waterloo Waterloo, ON Canada; 5 Department of Psychiatry and Behavioural Neurosciences McMaster University Hamilton, ON Canada

**Keywords:** adolescents, children, chronic disease, epidemiology, mental health, suicidality

## Abstract

**Background:**

Evidence suggests that having a chronic physical illness (CPI; eg, asthma, diabetes, and epilepsy) is an independent risk factor for suicidality (ie, suicidal ideation or attempts) among youth. Less is known about the mechanisms linking CPI and suicidality. Some evidence suggests that mental illness (eg, depression and anxiety) or neurodevelopmental disorder (eg, attention-deficit/hyperactivity disorder) mediates or moderates the CPI-suicidality association. Missing from the knowledge base is information on the association between having co-occurring CPI and mental illness or neurodevelopmental disorder (MIND) on youth suicidality.

**Objective:**

This study uses epidemiological data from the 2019 Canadian Health Survey of Children and Youth (CHSCY) to study the intersection of CPI, MIND, and suicidality in youth. We will estimate prevalence, identify predictors, and investigate psychosocial and service use outcomes for youth with CPI-MIND comorbidity versus other morbidity groups (ie, healthy, CPI only, and MIND only).

**Methods:**

Conducted by Statistics Canada, the CHSCY collected data from 47,850 children (aged 1-17 years) and their primary caregiving parent. Measures of youth CPI, MIND, family environment, and sociodemographics are available using youth and parent informants. Information on psychiatric services use is available via parent report and linkage to national administrative health data found in the National Ambulatory Care Reporting System and the Discharge Abstract Database, which allow the investigation of hospital-based mental health services (eg, emergency department visits, hospitalizations, and length of stay in hospital). Questions about suicidality were restricted to youths aged 15-17 years (n=6950), which form our analytic sample. Weighted regression-based analyses will account for the complex survey design.

**Results:**

Our study began in November 2023, funded by the American Foundation for Suicide Prevention (SRG-0-008-22). Access to the linked CHSCY microdata file was granted in May 2024. Initial examination of CHSCY data shows that approximately 20% (1390/6950) of youth have CPI, 7% (490/6950) have MIND, 7% (490/6950) seriously considered suicide in the past year, and 3% (210/6950) had attempted suicide anytime during their life.

**Conclusions:**

Findings will provide estimates of suicidality among youth with CPI-MIND comorbidity, which will inform intervention planning to prevent loss of life in this vulnerable population. Modeling correlates of suicidality will advance understanding of the relative and joint effects of factors at multiple levels—information needed to target prevention efforts and services. Understanding patterns of psychiatric service use is vital to understanding access and barriers to services. This will inform whether use matches need, identifying opportunities to advise policy makers about upstream resources to prevent suicidality. Importantly, findings will provide robust baseline of information on the link between CPI-MIND comorbidity and suicidality in youth, which can be used by future studies to address questions related to the impact of the COVID-19 pandemic and associated countermeasures in this vulnerable population of youth.

**International Registered Report Identifier (IRRID):**

DERR1-10.2196/57103

## Introduction

### Background

Suicide remains a leading global cause of mortality among youth aged 15-19 years [1], and findings show that the prevalence of suicide thoughts and attempts has increased from 14% to 18% and from 6% to 9%, respectively, in the past decade [2,3]. There is a disproportionate risk of suicidality in youth with risk factors grouping into several domains: sociodemographic and educational (male sex, sexual minority, low socioeconomic status, and low educational attainment), negative life events and family stressors (marriage dissolution, parental death, adverse childhood experiences, family history of mental illness, or suicidal behavior), and psychological and personality (mental or neurodevelopmental disorder, substance misuse, and hopelessness) [4-6]. There is also accumulating evidence that having a chronic physical illness (CPI; eg, asthma, diabetes, and epilepsy) is an independent risk factor for youth suicidality, with odds ratios (ORs) ≥2.0 compared with youth with no CPI in epidemiological studies [7-11]. Less is known about the mechanisms linking CPI and suicidality among youth. Some research shows abnormal hypothalamic-pituitary-adrenal axis functioning or increased disability [10,12] among those with CPI who have experienced suicidality. Some evidence suggests that mental illness, specifically major depression, mediates the association between CPI and suicidality [13], and other research has found mixed findings regarding the moderating effect of mental illness on the CPI-suicidality association [11,14].

### Rationale

Missing from the knowledge base is information on the association between having co-occurring CPI and mental illness or neurodevelopmental disorder (MIND) on suicidality among youth (or any other developmental group). Our forthcoming research in a small clinical sample of youth with CPI has shown increased suicidality among those with co-occurring MINDs. It is unknown the extent to which this preliminary finding can be extrapolated to the general population of youth. Other reports have adjusted for the presence of MIND when investigating suicidality among youth with CPI; however, such approaches do not allow for testing dose-response relationships. One study showed that youth with ≥2 disabilities had a higher likelihood of past-year suicide attempts (16%) than those with 1 (7%) or no disabilities (2%) [15]. Unfortunately, this work did not report the types of comorbidities. Furthermore, there is no information on psychosocial, academic, or substance use outcomes among youth with CPI-MIND comorbidity (or CPI only) who experienced suicidality.

Addressing knowledge gaps regarding the burden of suicidality among youth with CPI-MIND comorbidity and determinants and outcomes associated with suicidality, as well as use of psychiatric services, is desperately needed to inform the provision of timely and appropriate health services to reduce the incidence of suicidality in this vulnerable group. To do this, we need robust contemporary epidemiological data on a representative sample of youth. Our study meets these key criteria and is poised to maximize the impact of the findings. Importantly, information gained from this study can influence practice and policy in terms of best practices in the context of family-centered care and for ensuring successful transition from the pediatric to the adult health system. We describe the protocol for the first study to comprehensively investigate suicidality among youth with CPI-MIND comorbidity.

### Objectives and Hypotheses

This study uses epidemiological data from the 2019 Canadian Health Survey of Children and Youth (CHSCY) to study the intersection of CPI, MIND, and suicide ideation and attempts (herein suicidality) in youth. First, we will estimate the prevalence of suicidality among youth with CPI-MIND comorbidity. We hypothesize that youth with CPI-MIND comorbidity will have the highest prevalence of suicidality compared with other morbidity groups: CPI only, MIND only, and healthy controls (youth with no CPI or MIND). Epidemiological evidence of suicidality prevalence stratified by these morbidity groups is lacking; thus, we are unable to hypothesize specific prevalence estimates. However, we anticipate that likelihood of suicidality among youth will show a dose-response effect: CPI-MIND comorbidity > MIND only > CPI only > healthy youth.

Second, we will quantify magnitudes of association between CPI-MIND comorbidity and suicidality, adjusting for sociodemographic or economic factors. We hypothesize that the association between CPI-MIND comorbidity and suicidality will remain strong (OR ≥2.0) after covariate adjustment, indicating the persistence and importance of CPI-MIND comorbidity over and above other factors.

Third, we will identify multilevel correlates (individual, family, and neighborhood) of suicidality among youth with CPI-MIND comorbidity. We hypothesize that correlates of suicidality will include older age, male sex, transgender identity, increased levels of disability, parent psychopathology, strained parent-youth relationships, lower household income, and neighborhood disadvantage. We expect that these correlates will be similar to those found in other morbidity groups, although the magnitudes of association will be larger for those with CPI-MIND comorbidity, further highlighting their vulnerability.

Fourth, we will contrast patterns of psychiatric services use among youth with CPI-MIND comorbidity who have versus have not experienced suicidality. We hypothesize that youth with CPI-MIND comorbidity who experienced suicidality will have fewer psychiatric outpatient visits but more psychiatric inpatient and emergency department visits, as well as report more barriers to accessing services. Given the lack of research in this population of youth, we are unable to hypothesize whether associations will be similar to youth with CPI or MIND only.

Finally, we will test whether CPI-MIND comorbidity moderates associations between suicidality and health outcomes. Given the anticipated compounding effect of CPI-MIND comorbidity versus other morbidity groups, we hypothesize that suicidality will be associated with poorer psychosocial and academic outcomes and increased use of substances, and that these associations will be substantially larger for youth with CPI-MIND comorbidity.

## Methods

### Design and Sample

The CHSCY was conducted by Statistics Canada with data collected (in both official languages, English and French) between February and June 2019 and made available to researchers in July 2020 [16]. The target population was children and youth aged 1-17 years living in the 10 provinces and 3 territories. The sampling frame was the 2018 Canada Child Tax Benefit file, which covers approximately 98% of the target population. The study sample was selected by multistage, stratified, and random sampling of households within the Canada Child Benefit file. The sample is primarily stratified by province, with 2 exceptions: the 3 territories are grouped into a single stratum (“North”), and in Ontario (the most populous Canadian province), the subregions of the Administrative Health Regions within the province form the geographic strata.

From the identified households, there were a total of 47,850 participants aged 1-17 years who were included. Survey weights based on the probability of selection, adjusted for nonresponse and stratification, were created by Statistics Canada to ensure unbiased estimates and a representative sample. Field interviewers contacted households by telephone or in person to speak with the person most knowledgeable (PMK) about the household, described the study, confirmed eligibility, and invited eligible children and youth within the household to participate. The PMK provided self- and proxy-reported data for all children and youth in the study (aged 1-17 years). Youth aged 12-17 years were eligible to provide self-reported data (13,650/47,850, 29% of the CHSCY sample); however, measures of suicidality were asked only of youth aged 15-17 years; thus, we restrict our analyses accordingly (6950/47,850, 15% of the CHSCY sample) [17]. Sample proportions are based on rounded frequencies provided by Statistics Canada prior to data access and vetting of results [16]. Thus, it is reasonable to expect some differences in these values and those available in the master microdata file.

Participants were provided the opportunity to complete the survey on the web. If the e-survey was not completed by March 31, 2019, a Statistics Canada interviewer called and asked the participant to complete the survey over the telephone. The CHSCY also asked the participants to consent to the sharing of their personal information to facilitate data linkage with administrative health data (98% consented) [17].

### Ethical Considerations

Participant confidentiality is provided under the Statistics Act and data fidelity is guaranteed by Statistics Canada. Informed consent, collected by Statistics Canada, included the provision for secondary data analysis. All data in the analytic file that will be used by our research team are deidentified. As such, this study is exempt from ethics review by our institutional research ethics board in accordance with the Tri-Council Policy Statement: Ethical Conduct for Research Involving Humans (TCPS2), which states, “Research does not require [research ethics board] review when it relies exclusively on information that is publicly available through a mechanism set out by legislation or regulation and that is protected by law” [18].

We will access the CHSCY microdata file via the Canadian Research Data Centre Network, which is an example of information made available through legislation or regulation. According to the TCPS2, “Exemption from REB review for research involving this type of information is based on the presence of a custodian/steward designated in accordance with access to information and privacy legislation who protects privacy and proprietary interests associated with the information (eg, an access to information and privacy coordinator or a guardian of Canadian census data)” [18]. Our application to Statistics Canada to initiate this study was approved (#7327).

### Measures

The measures described will be used to address our research objectives. In asking about the physical and mental health of youth, the PMKs are instructed that “long-term conditions are those that are expected to last or have already lasted six months or more and that have been diagnosed by a health professional.”

#### Physical Health

CPIs in youth were assessed using a self-administered questionnaire. The PMK was asked, “Has this child been diagnosed with any of the following long-term conditions?” Binary responses were provided for the following CPIs: headache, allergies (including food, animal, dust mites, mold, pollens or grasses, chemicals, medicine, or other), hay fever or nasal allergy, asthma, diabetes, and epilepsy. The CHSCY also included PMK-reported height and weight of children, which was used to calculate BMI and subsequently used to classify youth with overweight or obesity according to the World Health Organization [17,19]. These conditions represent the most common CPIs affecting youth [20-23], and the estimated proportions in the CHSCY range from 1% to 20%. Furthermore, a final question asked to the PMKs was whether their children were diagnosed with “any other long-term physical, mental, developmental or intellectual conditions” (6%). Because this question aggregated CPIs and MINDs, the PMKs who positively endorse this question, but no other CPI, will be excluded from the analysis.

#### Mental Health

Using the same leading statement and response options for the assessment of CPI, MINDs in youth were ascertained by asking the PMK about the following: anxiety disorder (including phobia, obsessive-compulsive disorder, or panic disorder), mood disorder (including depression, bipolar disorder, mania, or dysthymia), attention-deficit/hyperactivity disorder, autism spectrum disorder (including autism, autistic disorder, Asperger disorder, or pervasive developmental disorder), and eating disorder (including anorexia or bulimia). The CHSCY also asks the PMKs about whether children have been diagnosed with fetal alcohol spectrum disorder or with a learning disability or disorder. These conditions represent the most common MINDs among youth [24,25], and the estimated proportions in the CHSCY range from 1% to 7%.

#### Suicidality

Suicidal ideation and attempts were measured by asking youth to respond to 2 standardized questions. These questions align closely with the suicidal ideation and attempt modules of the Self-Injurious Thoughts and Behaviors Interview, a gold standard instrument for assessing suicidality [26]. In the first question, youth were asked, “In the past 12 months, did you ever seriously consider attempting suicide or taking your own life?” In the second question, youth were asked, “Have you ever attempted suicide or tried taking your own life?” Estimates provided by Statistics Canada suggest that nearly 7% and 3% of youth aged 15-17 years positively endorsed the question on suicidal ideation and attempts, respectively.

#### Disability/Functioning

The CHSCY included the PMK-reported UNICEF/Washington Group child functioning module as a measure of youth disability across the broad domains of physical, behavior, communication, emotions, cognition, and social [27]. This module is psychometrically robust with a strong track record [28-30]. Evidence shows that constructs of functioning, disability, or pain are independently associated with poorer mental health in youth with CPI [31,32] and suicidality [11,13,14,33,34]. Thus, youth disability may confound the association between CPI-MIND comorbidity and suicidality and will be adjusted for in statistical models.

#### Psychosocial Health

Aligned with self-determination theory and adapted from the Intrinsic Need Satisfaction Scale [35], the CHSCY included 3 need subscales (autonomy, competence, and relatedness). Satisfaction with each need is assessed in 3 different contexts (home, school, and with friends), based on 6 items reported for the past week. Each item is a statement (eg, “I feel I have a choice about when and how to do my schoolwork” for autonomy, “I feel I do things well at home” for competence, and “My friends like me and care about me” for relatedness). Youth reported the extent to which the statements are true or false for them. Higher scores indicate higher levels of need satisfaction and are associated with youth well-being [35]. The CHSCY also included Statistics Canada standardized measures of perceived current life satisfaction using a 10-point scale, usual life stress using a 5-point scale, and usual level of happiness using a 5-point scale.

#### Education and Extracurricular Activities

Youth academic achievement was measured using an approximate overall grade for the current school year in which response options included percentages (eg, “80%-100%”) and associated letter grades (eg, “mainly A”). Youth were asked whether they had educational accommodations at school for the following: permanent physical disability; cognitive, behavioral, or emotional disability; gifted; or other. Past-week school absences and perceptions of how often youth look forward to going to school were assessed using a 4-point scale (never to always). Participation in extracurricular activities related to physical activities with a coach or instructor; music, drama, or art clubs or sessions; and other organized clubs or community groups was assessed using no or yes response options.

#### Victimization

Past-year bullying was asked of youth using a 5-point scale, which measured the frequency (never to daily) for 10 experiences (eg, made fun of, called names, or insulted; threatened with harm; pushed, shoved, tripped, or spit on; and hurtful information posted on the internet). A composite bullying score will be computed with higher scores indicating greater frequency of bullying.

#### Physical Activity and Substance Use

Statistics Canada generated derived variables to measure whether youth in the CHSCY adhered to the physical activity standards from the Canadian 24-hour movement guidelines [36-39] over the past week. Variables were derived for low physical activity and moderate to vigorous physical activity, which were dichotomized as meeting versus not meeting the Canadian guidelines.

Youth reported the frequency of their alcohol consumption over the past year (never, once a month or less, 2-3 times a month, and at least once a week) and frequency of past 30-day binge drinking. Statistics Canada also derived a variable that classified youth according to alcohol use (regular, occasional, did not drink in the past year, and never drinker). Questions are also asked regarding 30-day and lifetime cigarette smoking and a derived variable was computed (current or former smoker, experimental smoker, and never smoked). Also included is a measure of past 30-day e-cigarette use, which categorized youth according to use (<1 time per week, 2-3 times per week, 3-5 times per week, daily, or almost every day). Youth reports were also collected to measure frequency of past-year and past 3-month cannabis use (never, once or twice, 1-3 times per month, weekly, daily, or almost daily). The substance use measures are consistent with those derived from previously validated self-report measures appropriate for youth populations including other representative child and adolescent health surveillance studies in Canada [40-43], or measures specifically recommended by the Substance Use and Addictions Program at Health Canada. Consistent with previous research, the substance use measures can be used as either frequency or categorical measures.

#### Psychiatric Service Use

The PMKs were asked about whether their children required or received services for health-related concerns. We restrict our focus to the 3 health services listed in the CHSCY that are in the sphere of mental health care—focusing or controlling behavior, mental health, and learning. For those services that were endorsed by the PMK, questions were asked whether families experienced any of the following barriers to accessing each of the endorsed services: wait time too long, not available, cost, child not eligible, or other reason. The PMKs were also asked about required or received services from specific health professionals. In focusing on mental health professionals, we limit our analyses to “psychiatrists” and “psychologists or counselors” (as listed in the CHSCY). The same questions regarding barriers to accessing these mental health professionals were asked.

The CHSCY also collected individual health numbers of youth participants to link to databases to ascertain detailed health services use data [16,44]. These include the National Ambulatory Care Reporting System and the Discharge Abstract Database, which allow for the investigation of hospital-based mental health services (number of emergency department visits and hospitalizations, length of stay in hospital). As the data custodian, Statistics Canada will conduct the data linkages within their Social Data Linkage Environment and deposit the CHSCY-linked microdata file in our institutional Research Data Centre.

#### Parent and Family Health

The PMKs provided self-reports of perceived mental health using a 5-point scale, as well as life satisfaction and usual life stress using the same standardized questions described for youth. Canadian low-income cutoffs based on community size and number of individuals within the household will be used as a proxy of socioeconomic disadvantage [45].

#### Sex, Gender, and Sexual Attraction

Youth and their PMK reported on their individual (1) sex at birth and (2) current gender identity. Regarding gender identity, the CHSCY included the following for participants: “Gender refers to current gender which may be different from sex assigned at birth and may be different from what is indicated on legal documents.” Available response options are male, female, or please specify. Statistics Canada then used these 2 variables (sex at birth and current gender identity) to derive a variable for cisgender and transgender participants. This 2-step consideration of gender identity is the current gold standard [46]. Sexual minority status will be measured using youth and PMK-reported gender (male, female, and other) and sexual orientation using an instrument recommended by the Sexual Minority Assessment Research Team and included in the CHSCY [47]. Following previous work [48], we will construct the following sexual minority categories: heterosexual (only opposite gender attraction), boys who were only or mostly attracted to boys, girls who were only or mostly attracted to girls, those attracted to >1 gender (equally attracted to female and male individuals or mostly attracted to opposite gender), and unsure.

#### Community Factors

The CHSCY included an urban and rural area classification based on standardized thresholds for community size. This community-level characteristic will be used as a proxy for mental health service availability [49]. The PMKs are asked about whether their neighborhood is safe for children to play outside during the day (very unsafe to very safe). Neighborhood safety has been shown to influence child and adolescent health and health behaviors [50-53] but has not been used to examine differential effects for youth with versus with no CPI-MIND comorbidity.

#### Demographic Factors

Youth and their PMK reported on several demographic characteristics for themselves and their family, including, age, educational attainment, marital status, household income, number of people in the household, ethnicity, country of birth, and year of immigration.

### Analysis

Youth CPI-MIND comorbidity will be operationalized as the co-occurrence of ≥1 PMK-reported CPI and ≥1 PMK-reported mental illness. The complex design of the CHSCY requires that along with sampling weights, bootstrap weights be used to produce unbiased population estimates of variances for prevalence and model parameters, respectively [16]. Our approach will be to include youth and parent sex, gender identity, and sexual orientation as covariates in all our statistical models. If any of these variables are found to moderate any associations outlined in our research objectives, we will follow best practices [54], whereby sex- or gender-stratified models will be computed, reported, and interpreted to ensure that findings are broadly applicable.

Prevalence of suicidality across morbidity status (CPI-MIND comorbidity, CPI only, MIND only, and healthy controls) will be computed, along with associated 95% CIs for objective 1. The Rao-Scott *χ*^2^ test will assess whether prevalence differs across morbidity groups. Where possible, analyses will be conducted separately for suicidal ideation and suicide attempts, although we recognize that because the prevalence of suicide attempts is rare (3%), such investigations are likely to be underpowered and thus, exploratory.

Logistic regression, adjusting for relevant covariates, will be used to compute ORs and 95% CIs for the association between CPI-MIND comorbidity and suicidality (objective 2). Contrasts will be specified to determine whether ORs are different across morbidity groups. Covariates will be added to the unadjusted model sequentially in blocks that correspond to these levels: (1) youth: age, sex, gender identity, sexual attraction, immigrant, ethnicity, disability, victimization, and strained relationships with parents; (2) PMK: sex, gender identity, sexual attraction, education, immigrant, ethnicity, perceived mental health, and low-income household; and (3) community: size and neighborhood safety.

A multinomial logistic regression model will be used to address objective 3, correlates of youth suicidality with CPI-MIND comorbidity, whereby risk factors for morbidity groups will be compared with reference to healthy controls. Contrasts will be specified to determine whether ORs for statistically significant correlates of suicidality are different across morbidity groups—an important effort to identify morbidity-specific suicidality profiles among youth. Covariates from objective 2 will be added to the model in a single block as main effects. A second block will include product-term interactions that will be explored as potential moderators of the hypothesized associations between CPI-MIND comorbidity and suicidality.

To address objective 4, logistic regression models will be computed to examine potential differences in use of psychiatric services between youth with CPI-MIND comorbidity who have experienced suicidality and youth with CPI-MIND comorbidity who have not experienced suicidality. These logistic regression models will examine the independent outcomes of psychiatric service use or health professional consultations as listed in the CHSCY, as well as emergency department visits or hospitalizations for mental health as recorded in the National Ambulatory Care Reporting System and Discharge Abstract Database in the year prior to and the 2 years after the CHSCY. Poisson regression models will examine differences in the number of reported barriers for using psychiatric services, number of emergency department visits and hospitalizations, and associated lengths of stay in hospital between youth with CPI-MIND comorbidity who have versus have not experienced suicidality. Our approach follows definitions and codes used in the Mental Health and Addictions Scorecard and Evaluation Framework at the Institute of Clinical and Evaluative Sciences [55].

To determine whether CPI-MIND comorbidity moderates associations between suicidality and health outcomes in objective 5, product-term interactions will be included in a series of independent linear or logistic regression models. Outcomes include psychosocial health, physical activity, substance use, academic achievement, extracurricular activities, and victimization. In the presence of a statistically significant interaction, post hoc moderator analyses will be conducted to identify the direction of the effects [56].

### Study Power

The focus on youth aged 15-17 years suggests that an anticipated sample size (n=6950) will be available for analysis. Given the estimated proportions of suicidality and CPI-MIND comorbidity in the CHSCY, recent simulation studies for logistic and multinomial regression [57-60], as well as our calculations for linear regression [61], suggests that our proposed analyses are adequately powered at 1–β=.80 and α=.05 to address our research objectives. Specifically, *χ*^2^ tests used in objective 1 require a total sample size of 2268 to detect small differences across morbidity status (ω=0.1). Logistic regression models used to address objectives 2, 4, and 5 require a total sample size of 2262 to detect an OR of 2.0 for a main effect and a sample size of 6691 to detect an OR of 2.5 for product-term interactions. Similarly, linear regression models with ≤30 covariates will be adequately powered to detect very small effects (*f*^2^=0.01) with sample sizes of 1785 and 4195 for main and interaction effects, respectively. The multinomial regression model with unequal morbidity group sizes, 16 covariates, and 30 events per variable that will be used to address objective 4 requires a sample size of 5085. To maximize the analytical sample, an appropriate imputation technique, such as multiple imputation or full information maximum likelihood, will be used to account for data missing at random. Based on our previous experience working with epidemiological data in this field of study (which detected small to large effect sizes) [10,11,32,62-65], we expect that the sample size available in the CHSCY is sufficient.

## Results

Our study began in November 2023, funded by the American Foundation for Suicide Prevention (SRG-0-008-22), with linkage of the CHSCY to National Ambulatory Care Reporting System and Discharge Abstract Database in collaboration with Statistics Canada. Access to the CHSCY microdata file was made available to our research team in May 2024. Initial examination of CHSCY data shows that approximately 20% (1390/6950) of youth have CPI, 7% (490/6950) have MIND, 7% (490/6950) seriously considered suicide in the past year, and 3% (210/6950) had attempted suicide anytime during their life.

## Discussion

### Principal Outcomes

In this study, we will estimate the prevalence of suicidality among youth with CPI-MND comorbidity; quantify the magnitudes of association between CPI-MND comorbidity and suicidality, adjusting for relevant sociodemographic and economic factors; identify multilevel correlates of suicidality among youth with CPI-MND comorbidity; contrast patterns of psychiatric services use among youth with CPI-MND comorbidity who have versus have not experienced suicidality; and test whether CPI-MND comorbidity moderates associations between suicidality and health outcomes. Each of these objectives will be examined in reference to other morbidity groups (ie, healthy, CPI only, and MIND only) in the sample.

### Limitations

We anticipate some limitations to this study. First, the CHSCY relies upon PMK-reported diagnoses of a nonexhaustive list of CPIs and MINDs in youth. This can be problematic for chronic conditions with very low prevalence [66], leading to misclassification and attenuation of measures of association. However, evidence suggests that this bias is negligible [67]. Furthermore, the most common CPIs and MNDs affecting youth were included in the CHSCY, suggesting that coverage will be acceptable [20-25]. Concordance checks with diagnostic codes in the linked administrative health databases will also be conducted, if feasible. Second, given some evidence that associations between suicidality and CPIs may be heterogenous [9], we will test whether prevalence of suicidality across CPI-MIND comorbidities (eg, asthma-anxiety and diabetes-depression) is different. If this test is nonsignificant, then it would indicate support for the noncategorical approach to understanding suicidality among youth with physical-MND comorbidity [68,69]. The noncategorical approach posits—and has empirical evidence to support—that while differences in mental health or suicidality outcomes are large between youth with and with no CPI, differences across illnesses are relatively small [11,31,70]. Aggregating CPI-MIND comorbidities into a single variable will ensure model parsimony and statistical efficiency in addressing the research objectives. If the noncategorical approach is not supported, then CPI-MIND comorbidities will be included as dummy variable covariates in our models. Third, temporal ordering of study variables in relation to CPI and MIND onset, as well as suicidality, is limited in the CHSCY, thus preventing inferences on causality. We will explore opportunities to use PMK-reported age of diagnosis for physical and mental illnesses to untangle temporal associations; however, missing data for these variables are likely; thus, possible misclassification and subsequent bias induced by the timing of diagnoses are anticipated.

### Conclusions

The CHSCY is a large representative study and is the most contemporary national pre–COVID-19 pandemic data set in Canada. As a study led by Statistics Canada, the data are of the highest quality and primed for immediate use. Importantly, findings will provide a standardized baseline of information on the link between CPI-MIND comorbidity and suicidality in youth, which can be used by future studies to address questions related to the impact of COVID-19 and associated countermeasures in this vulnerable population. Furthermore, linking the CHSCY to national administrative health records provides an unprecedented opportunity to compare and contrast patterns of psychiatric service use among youth who have experienced suicidality across morbidity groups. Not only do such data provide accurate accounts of actual service use by avoiding recall biases associated with self-reported services use but linkage also permits the examination of potential barriers to psychiatric service use. There is a pressing need for this information to inform policies related to integrated physical-mental health services, as well as ensuring that supportive resources are available as youth transition from the pediatric to adult health system.

## Appendix

Multimedia Appendix 1 Peer review report from the American Foundation for Suicide Prevention.

## Abbreviations

CHSCY: Canadian Health Survey of Children and Youth (CHSCY)
CPI: chronic physical illness
MIND: mental illness or neurodevelopmental disorder
OR: odds ratio
PMK: person most knowledgeable
TCPS2: Tri-Council Policy Statement: Ethical Conduct for Research Involving Humans

## Data Availability

The microdata file used in this study is made available by Canadian Research Data Centre Network. An application for data access can be made on the web [71].
