# Supplementary material for: Suicidal Ideation and Attempts Among Youth With Physical-Mental Comorbidity in Canada: Proposal for an Epidemiological Study
Source: JMIR Res Protoc. 2024 Jul 4;13:e57103. doi: 10.2196/57103 (PMC11258520; doi:10.2196/57103)
Supplement: Multimedia Appendix 1 [file resprot_v13i1e57103_app1.pdf]

Mark Ferro, University of Waterloo; Ph.D.
Grant ID# SRG-0-008-22

Dear Dr. Ferro,

It is my great pleasure to inform you that your Grant proposal, "Suicidal Ideation and Attempts among Youth with Physical-Mental Comorbidity: A Pan-Canadian Study," in the amount of $124,499, has received favorable reviews. AFSP received 156 applications in the 2022 grant submission cycle; your grant scored a 2.35 on a scale of 1 (Excellent) to 9. **It was recommended to the Scientific Council for funding and approved by the Board of Directors.**

The reviewers expressed that this is an important study for suicide prevention. In addition, they noted that the team is strong and diverse, and the analyses make sense. They suggest that you look at those with suicidal ideation and those with suicide attempts both separately and together. Below is the detailed feedback.

Congratulations! You will receive a grant start-up packet soon with the goal of beginning funding by or before October 1, 2023. Please include your grant number, **SRG-0-008-22,** on all communications.

Sincerely,

Jill M Harkavy-Friedman PhD

Jill Harkavy-Friedman, PhD
Senior Vice President of Research

Reviewer Feedback

Significance

Strengths

In a comprehensive study, the team aims to utilize a vast database of population-based data to investigate the relationship between suicidality in young individuals and associated mental health issues, as well as other health problems, including neurodevelopmental disorders. They also have a variety of socio-economic factors available for analysis. This is a multi-faceted issue that needs unraveling to effectively target risks and related treatments, including preventive measures. This topic, which is less explored, holds potential to inform policy interventions and

healthcare service delivery, particularly for a higher risk group of young individuals with physical-MND comorbidities (PMC). The study will explore the dose-response relationship among PMC. This is a crucial area of research as the issue of suicide in young people with physical-MND comorbidities is understudied. This study will be the first to systematically investigate the associations between these factors using a substantial sample size. Unraveling the impact of physical and neurodevelopmental co-morbidities on suicide risk in youth is a significant goal of this research.

Weaknesses

This research is related to suicide ideation and behavior among individuals aged 15-17. The data comes from a cross-sectional survey, which means it captures a snapshot of the population at a particular time. However, because it's a cross-sectional study, it doesn't provide a clear timeline of when the diagnoses were made.

The study does note that all suicidal ideation and behavior occurred within the last 12 months, which could be seen as a limitation. It might be challenging to fully understand the precursor history or the progression of suicidal ideation and behavior over time due to this limitation.

Investigators

Strengths

The team possesses the necessary expertise in analyzing extensive public health datasets, bolstered by prior grant support and pre-existing work that scrutinizes physical and mental health within these datasets. Notably, they have previously received a grant from the American Foundation for Suicide Prevention (AFSP), indicating their successful track record in research. The principal investigator, an accomplished suicide researcher and trained biostatistician/epidemiologist, leads the team. The other members contribute their unique expertise, collectively forming a robust investigative team with a solid foundation for the successful completion of the proposed objectives.

Weaknesses

The work and funding for this project are primarily Canadian, with no additional support from other North American sources. The individual assigned as a data analyst for this work, who is being funded, possesses less experience compared to the other investigators involved in the project.

Innovation

Strengths

This study utilizes a public health dataset to investigate the origins of suicidal thinking and behavior, with a particular focus on youth who have physical, mental, and developmental health issues. It delves into the intersections and individual contributions of each aspect to suicidal thoughts and behaviors. This is the first population-based study on suicidal ideation associated with Physical, Mental, and Developmental (PMC) health issues, using a representative sample of the Canadian youth population. It marks the first time this unique survey is being employed to probe into suicide risk in this specific population. The novelty of this investigation lies in its systematic examination of the specific contributions of covariates and moderators to suicide thoughts and behaviors.

Weaknesses

This research utilizes an indirect method to investigate relationships with suicide, using a dataset that was initially created for other purposes. This approach primarily demonstrates potential associations rather than providing a comprehensive understanding of cause and effect. The sample for the study is derived from population survey data of youths aged 15-17, which raises questions about the applicability of the study's findings to a wider youth population. Additionally, the analytic strategies employed are relatively basic, and while a genuinely multi-predictor model approach might have been constrained by the low rates, innovative approaches have been suggested in the past to analyze small populations or low-rate events. However, these novel strategies are not discussed in this study.

Approach

Strengths

The researchers are planning to infer relationships within a large population-based dataset. They intend to use standard and suitable statistical models to answer the research questions using the available data. Their approach to utilizing the survey data is meticulous, accounting for survey weights and suggesting suitable statistical analyses. They propose to test the impact of potential risk and attenuating factors.

Weaknesses

This study focuses on 15–17-year-olds, uncovering associations but not causes. The health issues identified are based on surveys, not actual diagnoses, and may not represent all significant conditions for this age group. Key conditions like pediatric cancers are missing, potentially skewing results. The study's findings may be compromised due to the analysis of long-lasting physical and mental conditions. The presence of physical, mental, and psychosocial health conditions is likely interconnected. The use of an urban-rural dichotomy for mental health resource availability seems inappropriate. There may be confusion about the use of a multinomial logistic regression model, which should be a multilevel one. The use of count data models for counting outcomes isn't discussed. The proposed approach for Aim 5 could be misleading. There's no mention of plans to address survivor bias, a significant issue as

individuals with severe comorbidities may have passed away before age 15. The statistical analysis plan is unclear about the inclusion of moderators and interactions.

Environment

Strengths

The previous usage of this dataset is already supported, with costs identified, for both obtaining and analyzing it. This dataset is set within an excellent scientific environment, which ensures a successful completion of the proposed research.

Weaknesses

No significant weaknesses identified

Protection for Human Subjects

N/A

Data and Safety Monitoring Plan

N/A

Vertebrate Animals

N/A

Budget and Period of Support

The content does not require any changes. The budget and project duration are suitable for the accomplishment of all five specific goals. However, the timeline might be overly generous considering that data availability and merging won't pose any issues, allowing many of these analyses to be completed within the first year. The budget is sufficient but somewhat atypical, as the majority of costs are for securing data access and for a part-time data analyst. The data access costs are quite substantial, as evidenced by the attached quote from Statistics Canada, which states a cost of $55,375 USD for the linkage and resulting dataset for analysis. The total project cost, including taxes, is $63,585 as stated in the letter from the organization. It's important to note that the investigators are already receiving support from their respective departments.

Additional Comments to Applicant

N/A

Overall

Strengths

This innovative proposal could offer new insights into teen suicide, impacting policy and clinical practice. The research proposal centers on understanding teen suicide tendencies, especially in those with health conditions. It aims to study the link between suicidality and physical, mental, and chronic conditions among Canadian youth. The team, database, and extensive analysis of risk factors will be crucial for this study.

Weaknesses

The study delves into the causes of suicide; however, it does not immediately contribute to prevention or treatment strategies. The omission of physical conditions and the timing of suicide behaviors may hinder causal inference, which could potentially restrict the applicability of the study's findings. Moreover, the survey nature of the study does not permit longitudinal analysis, thus limiting the ability to thoroughly investigate causal effects.
